# Human dirofilariosis in Austria: the past, the present, the future

**DOI:** 10.1186/s13071-021-04696-4

**Published:** 2021-04-29

**Authors:** Katharina Riebenbauer, Philipp B. Weber, Julia Walochnik, Franz Karlhofer, Stefan Winkler, Sonja Dorfer, Herbert Auer, Julia Valencak, Martin Laimer, Alessandra Handisurya

**Affiliations:** 1 Department of Dermatology, Medical University of Vienna, Waehringer Guertel 18-20, 1090 Vienna, Austria; 2 Molecular Parasitology, Institute of Specific Prophylaxis and Tropical Medicine, Medical University of Vienna, Vienna, Austria; 3 Division of Infectious Diseases and Tropical Medicine, Department of Medicine I, Medical University of Vienna, Vienna, Austria; 4 Department of Dermatology and Allergology, University Hospital of the Paracelsus Medical University Salzburg, Salzburg, Austria

**Keywords:** Dirofilariosis, Nematode, *Dirofilaria repens*, *Dirofilaria immitis*, Austria, Eyelid

## Abstract

**Background:**

Dirofilariosis is a vector-borne parasitosis caused by filarial nematodes of the genus *Dirofilaria.* In humans, who represent accidental hosts, dirofilariosis is mostly caused by *Dirofilaria repens* and *Dirofilaria immitis*. In Austria, the first reported case occurred in 1978. Since then, several (case) reports have been published.

**Methods:**

A systematic and retrospective review of collected published cases and new, unpublished confirmed cases of human dirofilariosis occurring in Austria was performed. A nematode was extracted from the eyelid of a previously unreported case and subsequently characterized histologically and using molecular biology techniques.

**Results:**

Data on a total of 39 cases of human dirofilariosis in Austria occurring between 1978 and 2020 are summarized. Over the past four decades the incidence has markedly increased, in particular after 1998. Of the 39 patients, men and women were equally affected, and the mean age was 47.1 years. The area most frequently affected was the head (38.5% of cases). Confined ocular involvement was observed in 23.1% of cases, and nematodes were isolated from the neck/trunk, extremities and the genito-inguinal area in 25.6, 15.4 and 15.4% of patients, respectively. Microfilariae were detected in two cases. Of the 39 patients, only 73.9% tested positive for anti-filarial antibodies and 56.3% for eosinophilia, despite successful isolation of a nematode; consequently, these measures did not represent reliable markers for dirofilariosis. Most patients had a travel history to countries endemic for *Dirofilaria* species*.* One patient who had not traveled abroad represented the only autochthonous case recorded to date. *Dirofilaria repens* was the predominant species, identified in 89.7% of cases. In the newly reported case of subcutaneous dirofilariosis, a live non-gravid *Dirofilaria repens* adult female of 12 cm length was isolated from the eyelid of the patient, and a video of the extraction is provided.

**Conclusions:**

The incidence of human dirofilariosis cases has increased strikingly over the last four decades in Austria. More cases can be expected in the foreseeable future due to changes in human behavior and (travel) activities as well as climate changes and the associated alterations in the availability of the natural reservoir, the vectors and the intrinsic characteristics of the parasite.

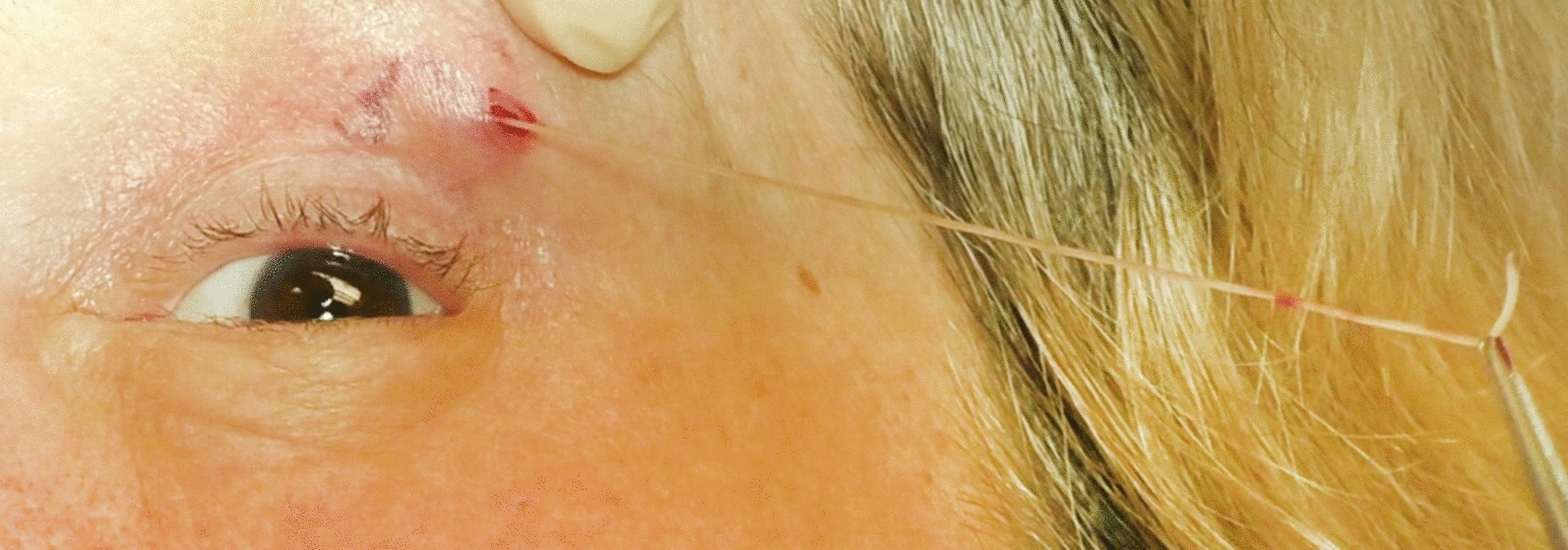

**Supplementary Information:**

The online version contains supplementary material available at 10.1186/s13071-021-04696-4.

## Background

Dirofilariosis is a vector-borne zoonosis caused by various species of filarial nematodes belonging to the genus *Dirofilaria*. The most important *Dirofilaria* species affecting humans are *D. repens* and *D. immitis*. Infections with other species, such as *D. tenuis*,* D. ursi*,* D. striata* and *D. subdermata*, are less common. In 2012, a new species with similarities to *D. repens* was identified in humans and dogs in Hong Kong and termed *D. hongkongensis* [1]. The most competent reservoirs of infection are domestic and wild canids (for *D. repens*, *D. immitis*), raccoons (for *D. tenuis*) and bears (for *D. ursi*). In the natural hosts the nematodes develop from third-stage larvae to adults. After copulation the female nematodes release microfilariae, which are taken up from the infected host’s peripheral bloodstream by blood-sucking arthropods, primarily mosquitoes of the genera *Aedes*,* Culex*,* Anopheles* and *Mansonia* [2–4]. These serve as intermediate hosts, in which development from microfilariae to the infective filariform larvae occurs, which are subsequently transmitted to a new host.

Humans represent aberrant hosts. *D. repens* typically causes human subcutaneous and ocular dirofilariosis, which is characterized by a migrating (pre-)adult worm that causes mild inflammatory reactions and/or nodules. In sporadic cases, nodules have been found in deeper organs, such as genitals, breasts, lungs, abdominal cavity and muscles [3, 4]. Infection with *D. immitis* can lead to human pulmonary dirofilariosis with the formation of nodules in the pulmonary arteries and occasionally at other ectopic sites, including subcutaneous tissues, eyes and internal organs [2, 5]. However, most dirofilarial infections resolve with mild symptoms or even remain unrecognized [2–5].

It has long been believed that *Dirofilaria* spp. cannot complete their life-cycle in humans and that infective larvae die shortly after transmission or at least fail to develop into sexually mature adults [4]. The situation, however, seems to be more complex, as in certain cases adult nematodes have been isolated from the affected patients, albeit in general only a single specimen was retrieved. Very rarely, microfilaraemia due to *Dirofilaria* spp. has been documented in humans [6–10].

The geographical distribution varies between the different *Dirofilaria* spp. [3, 11]. *D. repens* is prevalent in the Old World. In Europe, certain Mediterranean regions and countries, including Italy, southern France and Greece, as well as some central European countries, such as Hungary and the Republic of Serbia, are considered endemic regions. Increasing incidences of human dirofilariosis caused by *D. repens* have been reported in the past years in central and northern Europe, including the Ukraine, Russian Federation and Belarus, and transmissions were documented as far north as Finland and Siberia [12, 13]. The factors that influence the observed spread are multifaceted and complex [2, 4, 11] and include global climate changes and their impact on the life-cycle and activity of the vector as well as the global distribution of mosquito species due to international population movement, travel and trade. Also, the high prevalence of dirofilariosis in dogs, the major reservoir host, may contribute to the increasing infestations of humans. *D. immitis* is found worldwide, but particularly in the tropics and subtropics. Within Europe, the highest incidences of human dirofilariosis have been reported from southern countries, such as France, Greece, Italy, Portugal and Spain. In Austria, the first reported case of human dirofilariosis occurred in 1978 [14], and since then increasing numbers have been published, including the first, presumably autochthonous case in 2008 [15].

We report here our review of dirofilariosis in Austria in which we have collected and summarized data reported in all published and unpublished cases of human dirofilariosis in Austria from 1978 to 2020; report new, unpublished cases, including that of a patient with subcutaneous dirofilariosis from whom a live, adult *D. repens* was extracted from the eyelid and the procedure recorded; and provide a perspective on the potential drivers of this emerging disease.

## Methods

A systematic collection of published and unpublished cases of human dirofilariosis occurring in Austria from January 1978 to July 2020 was undertaken retrospectively. Data were extracted from publicly available sources (PubMed, Scopus) using the keywords “dirofilariosis,” “dirofilariasis,” “Dirofilaria,” “human” and “Austria,” as well as from internal databases of the Medical University of Vienna. Analyses were restricted to cases in which the diagnosis of human dirofilariosis or the presence of *Dirofilaria* spp. had been confirmed by histology, molecular biological techniques or serology. Information on the afflicted patient’s gender and age, localization of the lesions or the sites from which a nematode had been isolated, serological data (presence of anti-filarial antibodies, eosinophilia) at time of diagnosis, previous travel history and *Dirofilaria* spp. and characteristics was obtained.

For case number 38, serum samples were obtained at the time of diagnosis and at months 4 and 12 after extraction of the nematode and subjected to enzyme-linked immunosorbent assay (ELISA) to determine the presence of immunoglobulin G (IgG) against filarial antigens. The commercially available ELISA kit (Bordier Affinity Products SA, Crissier, Switzerland) based on antigens derived from the rodent filaria *Acanthocheilonema viteae* was employed according to the manufacturer’s instructions. This kit takes advantage of the antigenic homologies between human and the animal filariae and detects specific IgG against different filarial genera, including *Wuchereria*, *Brugia*, *Loa*, *Onchocerca* and *Mansonella*, with a reported sensitivity and specificity of 95 and 98%, respectively, but does not allow further identification of *Dirofilaria* to the species level. In order to detect potentially present microfilariae, blood was obtained at 8 p.m. at the 12-month time point and analyzed by membrane filtration. Briefly, whole blood was collected in EDTA, lysed with 10% Teepol 610 S (Sigma-Aldrich, St. Louis, MO, USA) and the mixture subsequently passed through a 5-µm pore-sized membrane filter (Nucleopore, Sigma-Aldrich) followed by several washing steps to remove the remaining blood. Finally, the filter was placed on a glass slide and stained with Giemsa. The nematode extracted from the eyelid was fixed in formalin and embedded in paraffin, following which 4-µm-thick tissue sections were cut, stained with hematoxylin–eosin for morphological analyses and images taken and digitalized using an Aperio slide scanner (Leica Biosystems, Nussloch, Germany). An approximately 0.5-cm-long piece of the nematode was homogenized and subjected to DNA isolation employing the QIAamp DNA Mini Kit (Qiagen, Hildesheim, Germany), following the manufacturer’s instructions. PCRs were performed using pan-filarial primers specific for the cytochrome* c* oxidase (*Cox*I) gene, namely Cox forward 5′-GCKTTTCCTCGTGTTAATGC-3′ and Cox reverse 5′-CCAGCCAAAACAGGAACAG-3′ primers, and confirmed with the pan-filarial primers FILf 5′-CGGTGATATTCGTTGGTGTC-3′ and FILr 5′-CTAGCTGCGTTCTTCATCGATC-3′ that amplify the internal transcribed spacer 1 (*ITS1*) of the ribosomal DNA [16, 17] at the following PCR conditions: initial denaturation at 95 °C,15 min; amplification at 95 °C/1 min, 56 °C/2 min, 72 °C/3 min for 30 cycles; a final extension at 72 °C for 7 min. Bands were extracted from agarose gels using the QIAquick® Gel Extraction Kit (Qiagen) and all amplicons were subjected to DNA sequencing. Sequences were obtained from both strands in two independent set-ups by direct sequencing in an automated ABI PRISM 310 Sequencer (PE Applied Biosystems, Langen, Germany) and assembled to consensus sequences using GeneDoc [18]. All consensus sequences were compared against reference sequences of *Dirofilaria* spp. available in GenBank by BLAST [19]. Multiple alignments were performed with ClustalX [20] and manually edited with GeneDoc [18] to exclude primer regions and to calculate identity scores. Sequence data were deposited in GenBank (accession numbers: MW617317 (*Cox*I) and MW617313 (*ITS1*).

Data on the average annual and the average summer temperatures as well as the average maximum temperatures in the summer months June/July/August in Austria over the period 1978–2020 were obtained from publicly available data of the Zentralanstalt für Meteorologie und Geodynamik (ZAMG), Austria (https://www.zamg.ac.at/cms/de/klima/klima-aktuell/klimamonitoring).

## Results

A total of 39 published and unpublished cases of human dirofilariosis occurring in Austria over the period January 1978 to July 2020 were collected; the results are summarized in Additional file 1: Table S1. For confirmation of the diagnosis or the presence of the *Dirofilaria* spp., histological analyses were performed in the majority (76.9%; 30/39) of cases. Data on anti-filarial antibodies are available since 1995; however, in 2006 PCR-based identification of the causative *Dirofilaria* spp. started to replace and/or support serology as the diagnostic technique (Additional file 1: Table S1).

**Fig. 1:**
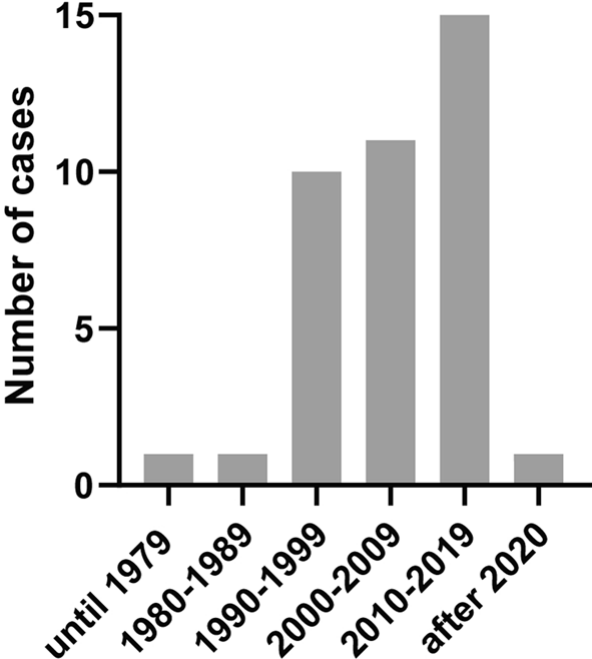
Cases of confirmed human dirofilariosis in Austria according to year of diagnosis

In the four decades since the first reported case in 1978 [14], there has been a steady increase in the incidence of human dirofilariosis in Austria (Fig. 1). Up to 1989 the number of cases of human dirofilariosis in Austria was very low, with one published case prior to 1980 [14] and one case from 1980 to 1989 [21]; in comparison, in the decade 1990–1999, a total of ten cases were reported [22–26]. The highest incidence of human dirofilariosis was observed in 1998, with four published cases. The numbers continued to rise, with a total of 11 cases from 2000 to 2009 [22, 25, 27, 28] and 15, partially new and unpublished cases from 2010 to 2019 [6, 17, 25, 29, 30].

**Fig. 2:**
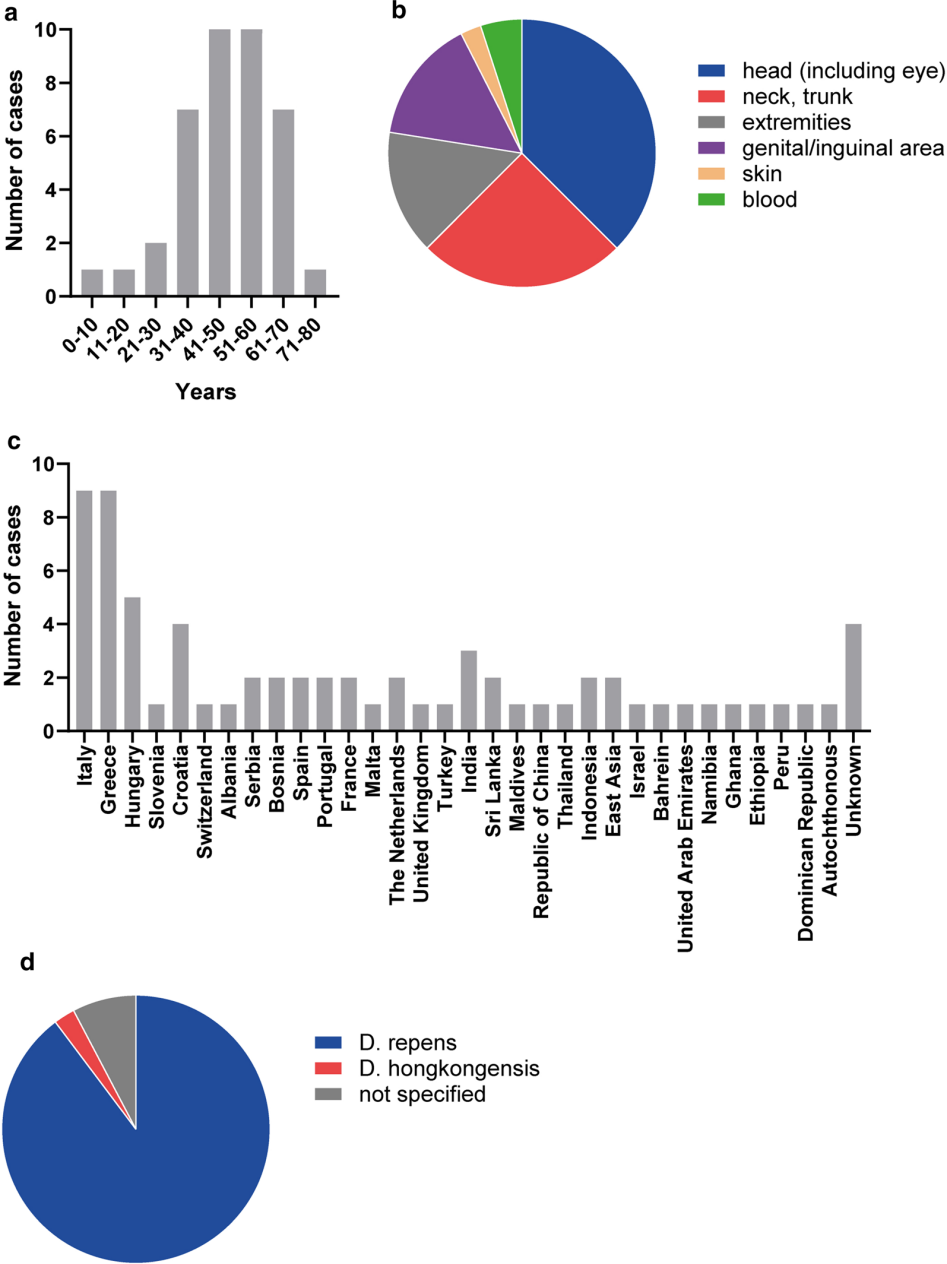
**a** Cases of confirmed human dirofilariosis in Austria by age. **b** Localizations of *Dirofilaria * species-induced lesions and sites from which a nematode had been isolated. **c** Travel history of patients with human dirofilariosis. **d** Causative *Dirofilaria* species identified in the cases of human dirofilariosis

Among the 39 Austrian patients comprising the study population, 48.7% (19/39) of the confirmed human dirofilariosis cases affected females and 51.3% (20/39) affected males. The patients’ ages ranged from 4 to 75 years (Fig. 2a), with a mean age of 47.1 years. Primarily adults in their fifth and sixth decade of life were affected, each constituting 25.6% (10/39) of the study population; 17.9% (7/39) of patients were in the 31- to 40-year age group and in the 61- to 70-year age group, respectively. In contrast, dirofilariosis was rarely found among children and adolescents (5.1%; 2/39). In the vast majority of the study population the nematode-induced lesions and the sites from where the respective worm had been isolated were confined to a distinct area of the body (Fig. 2b). It was mostly the head, including the eye, that was affected (38.5%; 15/39). Isolated involvement of the ocular region, such as the eyelid, eye, subconjunctival tissue and orbital cavity, was noted in 23.1% (9/39) of patients. The neck and trunk were similarly affected (25.6%; 10/39), and the extremities and genito-inguinal area were less involved (each at 15.4%; 6/39). From case no. 35, an adult *D. repens* was retrieved from a subcutaneous nodule located in the cervical region and microfilariae were subsequently detected in the same patient’s blood after membrane filtration by PCR [6]. Microfilariaemia was absent from the remaining cases, with the exception of case no. 36, where a single microfilaria was isolated from the patient’s blood after membrane filtration. PCR and DNA sequencing performed on this material revealed *D. repens*. In case no. 18, involvement of the skin without specification of the exact localization was reported [25]. Data on anti-filarial antibodies and eosinophil counts were available for 59.0% (23/39) and 41.0% (16/39) of the study population, respectively. Despite successful extraction of a worm in most of these cases, anti-filarial antibodies were detectable in only 73.9% (17/23) of patients and eosinophilia in 56.3% (9/16) of patients with modest elevation ranging from 6 to 27%. The highest rate of anti-filarial antibodies was 27% (total leucocyte count of 6.170 g/l), observed in case 36, where the patient also had microfilaraemia [6]. Travel history was obtained for 89.7% (34/39) of the study population; for the remaining 10.3% (4/39) of patients, the travel history was unknown (Fig. 2c). The majority of afflicted patients had frequently traveled within Europe (61.5%; 24/39) and, in particular, had traveled to Mediterranean countries and Hungary where *Dirofilaria* spp. are considered endemic: 30.8% (12/39) had visited one or more neighboring countries of Austria, including Italy, Slovenia, Hungary, Slovakia, Czechia, Germany, Switzerland and Liechtenstein; 25.6% (10/39) and 10.3% (4/39) had traveled to destinations in Asia and Africa, respectively. The patient described as case no. 20 was confirmed to have never traveled outside of Austria and is regarded as the only autochthonous Austrian case of human dirofilariosis to date [15].

The predominant *Dirofilaria* spp. identified in the study population was *D. repens*, representing the causative agent in 89.7% (35/39) of cases (Fig. 2d). Case no. 18, although serologically positive for *D. immitis*, was assumed to be caused by *D. repens*, as the infection had most probably been acquired in Peru and the observed subcutaneous wandering nodules were indicative of the latter [25]. Infection with *D. hongkongensis* was reported in 2.6% (1/39) [17] of the study population; the species was not identified in 7.7% (3/39) of the cases.

**Fig. 3:**
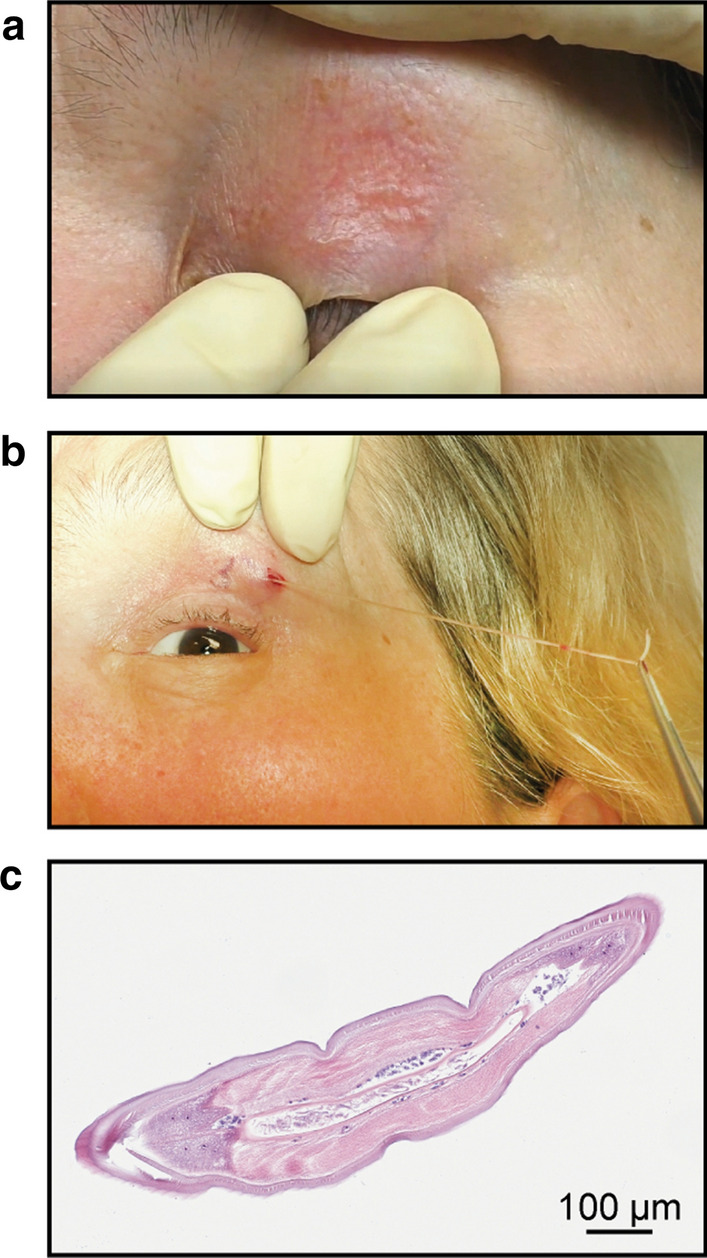
**a** A long, winded, palpable, subcutaneous structure on the erythematous, left upper eyelid of a 64-year old woman who complained of recurrent creeping sensations. **b** Extraction of a 12-cm-long, live nematode. **c** Hematoxylin–eosin stained section of the adult, female nematode belonging to genus *Dirofilaria*. Scale bar is depicted

In February 2019, a 64-year old woman of Bosnian origin (case 38) presented with creeping sensations in her right eye and eyelid. On inspection, a mild eczema of the right eyelid was noted, otherwise eye and skin were inconspicuous, and no tumor mass or edema were observed. High-frequency ultrasound examination of the orbital and ocular structures revealed mild inflammation, but no motile, worm-like structures, and the eyelid and the retro- and parabulbar orbita were without any pathology. Test results from routine blood analyses, including eosinophil counts and serum IgE levels, were within normal reference ranges. The patient has been living in Vienna, Austria, for many decades. She reported having spent the summer of 2018 in her house located in a rural region of Bosnia and recalled having been bitten by mosquitoes several times. Based on her history, the tentative diagnosis of subcutaneous dirofilariosis was made. One week later the patient presented again with recurrent creeping sensations, this time affecting her left upper eyelid and the left side of her neck. Physical examination revealed an erythematous left eyelid and a long, winded, palpable, subcutaneous structure (Fig. 3a). After local anesthesia with prilocaine, a 3-mm-long incision of the overlying skin of one blind end of the structure on the upper left eyelid was made and a 12-cm-long, live nematode was carefully extracted *in toto* (Fig. 3b)*.* A video of the extraction procedure is provided as Additional file 2. The nematode was identified by histology (Fig. 3c) as an adult, non-gravid female *Dirofilaria* spp.; subsequent PCR and DNA sequencing revealed 99% sequence identity in *Cox*I to various isolates of *D. repens*. However, the filarial serology was positive prior to extraction of the nematode and remained positive throughout the observation period of 1 year. Microfilariae were not detectable in the patient’s nocturnal peripheral blood; however, data were only available from the 12-month follow-up time point.

## Discussion

The numbers of reported human dirofilariosis cases in Austria have risen considerably in the past four decades. However, despite a thorough collection of the available data, the incidence is likely to be underestimated due to the lack of a mandatory and centrally coordinated reporting system and misdiagnosed and unrecognized cases. This raises the concern that dirofilariosis will emerge as an endemic zoonotic parasitosis throughout Europe in the near future. In this context, an increase in human dirofilariosis cases has been reported throughout central and northern Europe, in particular in the Russian federation, Belarus and Ukraine, but also in Hungary, Bulgaria and Slovakia [2, 14, 31–35]. In addition, autochthonous cases of dirofilariosis in humans have been reported in countries neighboring Austria, such as Czechia, Germany, Hungary and Slovakia [36–40].

The dissemination of *Dirofilaria* spp. in a certain region depends on several factors, including the availability of reservoir hosts, presence of the vectors, climate (which in turn affects mosquito density and the development of *Dirofilaria* inside the vector) and changes in human behavior and activity [2, 4].

A recent study on canine dirofilariosis conducted in Austria revealed that the number of dogs infected with *D. repens* and *D. immitis* has increased since 2014, reaching a peak in 2018 [41]. The infected animals had mostly been imported as shelter or stray dogs by animal welfare organizations or had an international travel history, both facilitated by the Pet Travel Scheme (Regulation [EU] No. 576/2013 of the European Parliament and of the Council as of 2013). In the majority of cases, the infected dogs were relocated from or had traveled to regions known to be endemic for *Dirofilaria* spp., such as Hungary, Greece, Croatia, Serbia, Slovenia, Spain, Romania and Slovakia [25, 41–47]. Dogs are the main reservoir hosts for *Dirofilaria* spp. and can easily introduce these parasites to previously non-endemic countries as competent vectors are available almost worldwide. However, in several of the cases presented herein, no travel history was traceable, suggesting the occurrence of autochthonous canine infections [25, 41, 42]. In contrast, autochthonous human dirofilariosis is still rare in Austria; to date, there has been only one presumably autochthonous case with the patient reporting no travel activities [15]. However, as the affected patient was employed as a border official in eastern Austria, the exact allocation of the causative infected mosquito to Austria or to the neighboring country of Hungary, which is known to be endemic for *Dirofilaria* spp. remains unresolved.

Furthermore, human migratory activities have increased in the past decades. For example, in 2019 around 9,884,000 main vacation trips with at least four overnight stays were documented for Austrian residents; of these, approximately 6,662,000 trips had international destinations [48]. In comparison, in 1978 only 3,682,000 main vacation trips were recorded, of which only one half had an international destination. Italy and Greece in particular were favorite destinations of the Austrian population, and both of these countries are endemic for dirofilariosis; specifically, about 19–33% and 4–12% of all main vacations between 1978 and 2018 were spent in these countries, respectively. This is also reflected by our study population, with the majority reporting international travels. While it can be assumed that the majority of cases diagnosed in Austria represent imported cases, future case–control studies are warranted to unequivocally prove this assumption.

**Fig. 4:**
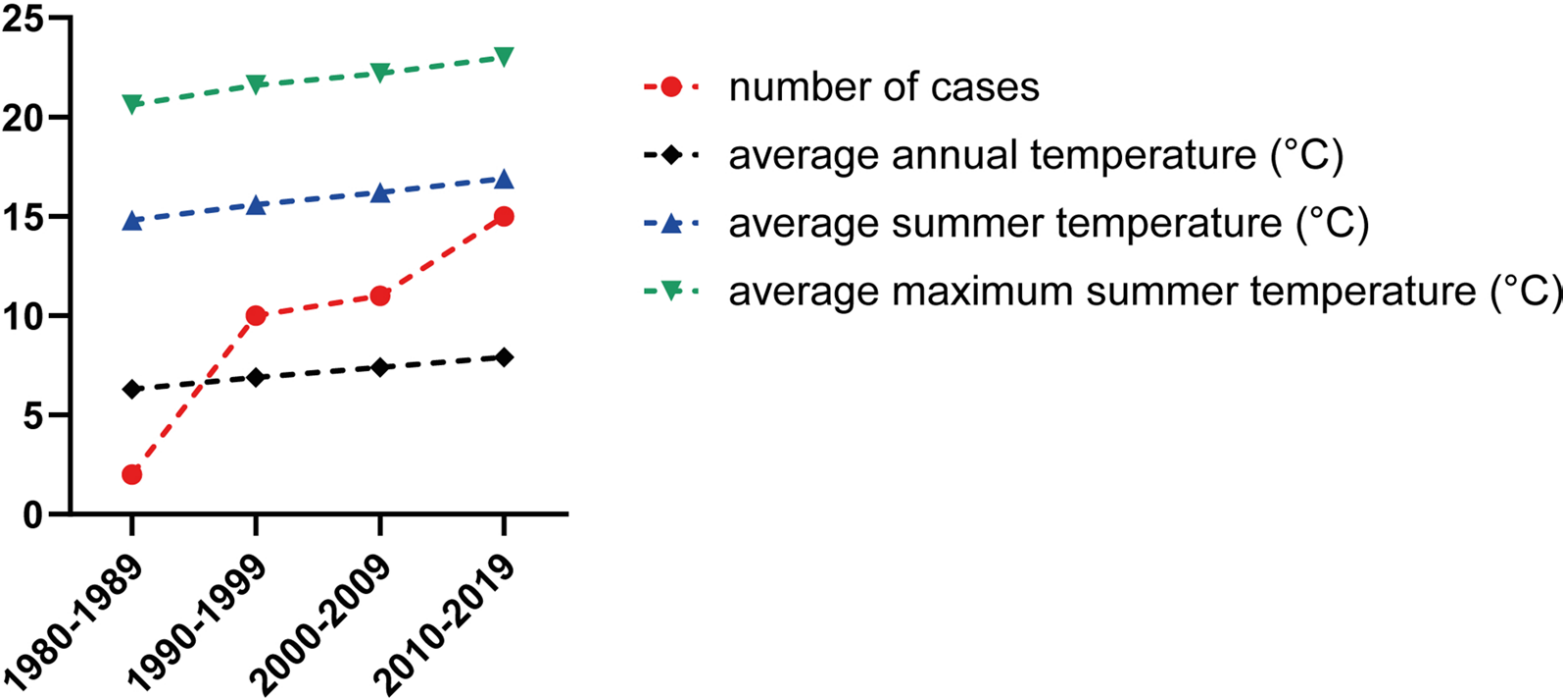
Number of human dirofilariosis cases in Austria by year of diagnosis (red dashed line), average annual temperature (in °C; black dashed line), average summer temperature (in °C; blue dashed line) and average maximum temperature during the summer in Austria (in °C; green dashed line)

The spread of mosquito species, which function as competent vectors for *D. repens* and *D. immitis*, could have contributed to the rising numbers of human dirofilariosis cases in Austria [25]. Autochthonous findings of *D. repens* in mosquitoes trapped in the eastern parts of Austria during a nationwide mosquito monitoring and surveillance program have already been reported [49]. Globalization, such as international trade activities and modern modes of transportation, facilitate the introduction of new vector species, such as *Aedes albopictus* (Asian tiger mosquito), *Ae. koreicus* or *Ae. japonicus* to Europe [11, 50–52]. Another major factor responsible for the increasing abundance and diversity of mosquito vectors lies within the context of global climate changes. Rising temperatures not only prolong the period of mosquito activity, but also accelerate the development of the *Dirofilaria* larvae within the vector [40, 53]. The developmental process strongly depends upon the surrounding temperatures, requiring 8–13 days at 27–30 °C and 16–20 days at 22 °C, stopping completely at temperatures of 14 °C or lower [2, 3]. Accordingly, data collected from the ZAMG (Fig. 4) demonstrated a noticeable, gradual increase in the average annual temperature from 6.3 °C in the period 1980–1989 up to 7.9 °C in the period 2010–2019 in Austria. Similarly, the average temperatures in summer rose by 2.9 °C from 14.8 °C to 16.9 °C between the periods 1980–1989 and 2010–2019. Of the 10 years with the highest temperatures in Austria since the beginning of record-keeping in 1768, 9 have been in the 21st century, as stated in the latest climate status report of the Climate Change Centers Austria [54], and the years 2018, 2014 and 2019 represent the three warmest years ever recorded in Austria. Hence, the temperatures in certain, primarily eastern, parts of Austria have already reached the 130 Dirofilaria Development Units (degree-days above 14 °C) necessary for the microfilariae to evolve to the infective stage in mosquitoes [55]. Furthermore, urbanization with formation of urban heat islands (UHIs) has an impact on the ambient climate. The phenomenon of UHIs is explained by the fact that dark surfaces, such as concrete, tend to store warmth and release it slowly and therefore lead to local urban microenvironments with higher temperatures. These heat units could support the diversity as well as the (year-round) survival of vectors and ultimately the presence of *Dirofilaria* spp.

Our study population showed a slight preponderance of human dirofilariosis in males and in adults in their fifth and sixth decade of life, whereas in other countries afflicted patients were more often female and of a younger age (range 21–40 years) [32, 56]. The reasons for the observed differences are not clear. One possible explanation might lie within population-specific or national differences in daily activities and habits, including outdoor and international travel activities or clothing preferences, or the discrepancies might merely be caused by the small size of our study population and the possibility of missed cases due to the lack of a central reporting system. Dirofilariosis in minors in Austria was rare, which is similar to the numbers reported in other countries, wih the exception of Sri Lanka, where more than one third of human dirofilariosis cases were found among children and adolescents [2, 57, 58]. Consistent with previous reports [2, 32], the most frequent affected localization in our study population was the upper half of the body, particularly the head. Presumably, the nematodes tend to stay in the vicinity of the mosquito bite, which is more likely to occur in areas of uncovered skin, such as the face, neck and arms, or, alternatively, symptoms are more easily detectable in these regions. The vast majority of the Austrian cases were caused by *D. repens*, which coincides with the observation that *D. repens* is spreading faster throughout Europe than *D. immitis* [3, 4, 11]. Adult worms were isolated in the majority of our cases, possibly due to the low awareness of dirofilariosis in Austria and the paucity of symptoms after infection with *Dirofilaria* spp.; hence, the amount of time elapsing until diagnosis gives the nematodes the time to mature. The lack of reliable, specific and sensitive serological tests further impairs diagnosis in humans. The results of this study on the presence of anti-filarial antibodies and eosinophilia in confirmed human *Dirofilaria* cases corroborate that the diagnostic values of both measures are rather limited.

Several (additional) key issues need to be addressed, as more cases of human dirofilariosis can be expected in the future. The development of more specific and sensitive diagnostic tools for the most important species, *D. repens* and *D. immitis*, for animals and humans is urgent. This would enable the screening of potentially infected pets and also of humans. The awareness of physicians, veterinarians as well as pet owners about the diseases should be raised, not only regarding human dirofilariosis, but also canine dirofilariosis, particularly in central Europe. Regular evaluations of the dirofilarial status in dogs and cats should be implemented, particularly after travel to endemic areas. Animals imported from endemic areas should be thoroughly examined for dirofilariosis by experienced veterinarians and chemoprophylaxis with macrocyclic lactones implemented as needed [59]. Furthermore, the measures for prevention and treatment recommended by the European Society of Dirofilariosis and Angiostrongylosis (ESDA) should be communicated and applied [59, 60] in order to decrease the risk for transmission to humans. To halt the expanding geographic distribution of both the vectors and the pathogens, effective measures to control global climate changes need to be taken, especially in light of the prediction of the Intergovernmental Panel on Climate Change of a further increase in temperatures of between 0.3 °C and 0.7 °C until 2035 [61].

## Conclusions

The incidence of human dirofilariosis in Austria has increased in the past decades and more cases can be expected in the near future. Changes in human behavior and (travel) activities as well as global climate changes and associated alterations in the availability of reservoir hosts and competent vectors may constitute the responsible key factors. While in this study trends for an association between these drivers and the rising of dirofilariosis in Austria are demonstrated, further research on the relationship between this disease and the (micro-)climatic conditions, mosquito vectors ecology and afflicted patients’ characteristics and habits, including migratory activities, are urgently warranted.

## Supplementary Information

**Additional file 1: Table S1.** Confirmed cases of human dirofilariosis in Austria.

**Additional file 2.** Movie file showing the complete extraction of the *Dirofilaria repens* nematode from the eyelid.

## Data Availability

All data generated or analyzed during this study are included in this published article and its additional files.

## Abbreviations

D.: Dirofilaria
spp.: Species
no: Number
PCR: Polymerase chain reaction
*Cox*1: Cytochrome* c* oxidase subunit I gene
ITS1: Internal transcribed spacer 1
ESDA: European Society of Dirofilariosis and Angiostrongylosis
ZAMG: Zentralanstalt für Meteorologie und Geodynamik
UHI: Urban heat islands
ESDA: European Society of Dirofilariosis and Angiostrongylosis
IPCC: Intergovernmental Panel on Climate Change

## References

[1] To KKW, Wong SSY, Poon RWS, Trendell-Smith NJ, Ngan AHY, Lam JWK (2012). A novel *Dirofilaria* species causing human and canine infections in Hong Kong. J Clin Microbiol.

[2] Simón F, Siles-Lucas M, Morchón R, González-Miguel J, Mellado I, Carretón E (2012). Human and animal dirofilariasis: the emergence of a zoonotic mosaic. Clin Microbiol Rev.

[3] Capelli G, Genchi C, Baneth G, Bourdeau P, Brianti E, Cardoso L (2018). Recent advances on *Dirofilaria repens* in dogs and humans in Europe. Parasites Vectors.

[4] Genchi C, Kramer L (2017). Subcutaneous dirofilariosis (*Dirofilaria repens*): an infection spreading throughout the old world. Parasites Vectors.

[5] Pampiglione S, Rivasi F, Gustinelli A (2009). Dirofilarial human cases in the Old World, attributed to *Dirofilaria immitis*: a critical analysis. Histopathology.

[6] Lechner AM, Gastager H, Kern JM, Wagner B, Tappe D (2020). Case report: successful treatment of a patient with microfilaremic dirofilariasis using doxycycline. Am J Trop Med Hyg.

[7] Nozais JP, Bain O, Gentilini M (1994). A case of subcutaneous dirofilaria (Nochtiella) repens with microfilaremia originating in Corsica. Bull Soc Pathol Exot.

[8] Petrocheilou V, Theodorakis M, Williams J, Prifti H, Georgilis K, Apostolopoulou I (1998). Microfilaremia from a *Dirofilaria*-like parasite in Greece. Case report APMIS.

[9] Kłudkowska M, Pielok Ł, Frąckowiak K, Masny A, Gołąb E, Paul M (2018). Dirofilaria repens infection as a cause of intensive peripheral microfilariemia in a Polish patient: process description and cases review. Acta Parasitol.

[10] Potters I, Vanfraechem G, Bottieau E (2018). *Dirofilaria repens* Nematode infection with microfilaremia in traveler returning to Belgium from Senegal. Emerg Infect Dis.

[11] Genchi C, Kramer LH (2020). The prevalence of *Dirofilaria immitis* and *D. repens* in the old world. Vet Parasitol.

[12] Pietikäinen R, Nordling S, Jokiranta S, Saari S, Heikkinen P, Gardiner C (2017). *Dirofilaria repens* transmission in southeastern Finland. Parasites Vectors.

[13] Kartashev V, Tverdokhlebova T, Korzan A, Vedenkov A, Simón L, González-Miguel J (2015). Human subcutaneous/ocular dirofilariasis in the Russian Federation and Belarus, 1997–2013. Int J Infect Dis.

[14] Bardach H, Heimbucher J, Raff M (1981). Subkutane *Dirofilaria* (Nochtiella) repens-Infektion beim Menschen-Erste Fallbeschreibung in Osterreich und Ubersicht der Literatur. Wien Klin Wochenschr.

[15] Auer H, Susani M (2008). Der erste autochthone Fall einer subkutanen Dirofilariose in Österreich. Wien Klin Wochenschr.

[16] Koehsler M, Soleiman A, Aspöck H, Auer H, Walochnik J (2007). Onchocerca jakutensis filariasis in humans. Emerg Infect Dis.

[17] Winkler S, Pollreisz A, Georgopoulos M, Bago-Horvath Z, Auer H, To KKW (2017). Candidatus *Dirofilaria hongkongensis* as causative agent of human ocular filariosis after travel to India. Emerg Infect Dis.

[18] Nicholas KB, Nicholas HBJ, Deerfield DW (1997). GeneDoc: analysis and visualization of genetic variation. EMBNEW NEWS.

[19] Altschul SF, Gish W, Miller W, Myers EW, Lipman DJ (1990). Basic local alignment search tool. J Mol Biol.

[20] Thompson JD, Gibson TJ, Plewniak F, Jeanmougin F, Higgins DG (1997). The CLUSTAL_X windows interface: flexible strategies for multiple sequence alignment aided by quality analysis tools. Nucleic Acids Res.

[21] Lammerhuber LC, Auer H, Bartl G, Dressler H (1990). Subkutane Dirofilaria (Nochtiella) repens-Infektion im Oberlidbereich. Spektrum Augenheilkd.

[22] Auer H (2004). Die Dirofilariose des Menschen-Epidemiologie und Nosologie einer gar nicht so seltenen Parasitose in Österreich (Nematoda, Spirurida, Onchocercidae). Denisia.

[23] Schuller-Petrovic S, Kern TH, Hassl A, Hermentin K, Gebhart W (1996). Subcutaneous dirofilariasis in man—case report from Austria. H G Z Hautkrankh..

[24] Braun H, Koele W, Stammberger H, Ranner G, Gröll R (1999). Endoscopic removal of an intraorbital "tumor": a vital surprise. Am J Rhinol.

[25] Fuehrer HP, Auer H, Leschnik M, Silbermayr K, Duscher G, Joachim A (2016). Dirofilaria in humans, dogs, and vectors in Austria (1978–2014)—from imported pathogens to the endemicity of *Dirofilaria repens*. PLoS Negl Trop Dis.

[26] Auer H, Weinkammer M, Bsteah A, Schnayder C, Dietze O, Kunit G (1997). Ein seltener Fall einer *Dirofilaria repens*-Infestation des Nebenhodens. Mitt Österr Ges Tropenmed Parasitol.

[27] Böckle BC, Auer H, Mikuz G, Sepp NT (2010). Danger lurks in the Mediterranean. Lancet.

[28] Auer H, Aspöck H (2010). Dirofilariosen des Menschen–seltene Helminthozoonosen auch in Mitteleuropa (Nematoda, Spirurida, Onchocercidae). Denisia.

[29] Ritter A, Egger S, Emesz M (2012). Dirofilariosis: subconjunctival infection with *Dirofilaria repens*. Ophthalmologe.

[30] Haim A, Kitchen M, Auer H, Rettenbacher T, Schmuth M (2020). A case of human *Dirofilaria repens* infection, causing an asymptomatic subcutaneous nodule. Parasitol Res.

[31] Kondrashin AV, Morozova LF, Stepanova EV, Turbabina NA, Maksimova MS, Morozov EN (2020). Anthology of *Dirofilariasis* in Russia (1915–2017). Pathogens.

[32] Sałamatin RV, Pavlikovska TM, Sagach OS, Nikolayenko SM, Kornyushin VV, Kharchenko VO (2013). Human dirofilariasis due to *Dirofilaria repens* in Ukraine, an emergent zoonosis: epidemiological report of 1465 cases. Acta Parasitol.

[33] Dóczi I, Bereczki L, Gyetvai T, Fejes I, Skribek Á, Szabó Á (2015). Description of five dirofilariasis cases in South Hungary and review epidemiology of this disease for the country. Wien Klin Wochenschr.

[34] Szénási Z, Kovács AH, Pampiglione S, Fioravanti ML, Kucsera I, Tánczos B (2008). Human dirofilariosis in Hungary: an emerging zoonosis in central Europe. Wien Klin Wochenschr.

[35] Velev V, Vutova K, Pelov T, Tsachev I (2019). Human *Dirofilariasis* in Bulgaria between 2009 and 2018. Helminthologia.

[36] Gebauer J, Ondruš J, Kulich P, Novotný L, Sałamatin R, Husa P (2021). The first case of periorbital human dirofilariasis in the Czech Republic. Parasitol Res.

[37] Tappe D, Plauth M, Bauer T, Muntau B, Dießel L, Tannich E (2014). A case of autochthonous human Dirofilaria infection, Germany, March 2014. Euro Surveill.

[38] Babal P, Kobzova D, Novak I, Dubinsky P, Jalili N (2008). First case of cutaneous human dirofilariosis in Slovak Republic. Bratisl Lek Listy.

[39] Matějů J, Chanová M, Modrý D, Mitková B, Hrazdilová K, Žampachová V (2016). Dirofilaria repens: emergence of autochthonous human infections in the Czech Republic (case reports). BMC Infect Dis.

[40] Genchi C, Kramer LH, Rivasi F (2011). Dirofilarial infections in Europe. Vector Borne Zoo Dis.

[41] Sonnberger K, Duscher GG, Fuehrer HP, Leschnik M (2020). Current trends in canine dirofilariosis in Austria—do we face a pre-endemic status?. Parasitol Res.

[42] Duscher G, Feiler A, Wille-Piazzai W, Bakonyi T, Leschnik M, Miterpakova M (2009). Detection of *Dirofilaria* in Austrian Dogs. Berl Munch Tierarztl Wochenschr.

[43] Széll Z, Bacsadi Á, Szeredi L, Nemes C, Fézer B, Bakcsa E (2020). Rapid spread and emergence of heartworm resulting from climate and climate-driven ecological changes in Hungary. Vet Parasitol.

[44] Farkas R, Mag V, Gyurkovszky M, Takács N, Vörös K, Solymosi N (2020). The current situation of canine dirofilariosis in Hungary. Parasitol Res.

[45] Tasić-Otašević SA, Trenkić Božinović MS, Gabrielli SV, Genchi C (2015). Canine and human *Dirofilaria* infections in the Balkan Peninsula. Vet Parasitol.

[46] Diakou A, Kapantaidakis E, Tamvakis A, Giannakis V, Strus N (2016). Dirofilaria infections in dogs in different areas of Greece. Parasites Vectors.

[47] Miterpáková M, Antolová D, Ondriska F, Gál V (2017). Human Dirofilaria repens infections diagnosed in Slovakia in the last 10 years (2007–2017). Wien Klin Wochenschr.

[48] Statistik Austria. Reisegewohnheiten. 2020. https://www.statistik.at/web_de/statistiken/wirtschaft/tourismus/reisegewohnheiten/index.html. Accessed 22 Oct 2020.

[49] Silbermayr K, Eigner B, Joachim A, Duscher GG, Seidel B, Allerberger F (2014). Autochthonous *Dirofilaria repens* in Austria. Parasites Vectors.

[50] Montarsi F, Ciocchetta S, Devine G, Ravagnan S, Mutinelli F, Frangipane di Regalbono A (2015). Development of Dirofilaria immitis within the mosquito Aedes (Finlaya) koreicus, a new invasive species for Europe. Parasites Vectors.

[51] Montarsi F, Martini S, Michelutti A, Da Rold G, Mazzucato M, Qualizza D (2019). The invasive mosquito *Aedes japonicus* japonicus is spreading in northeastern Italy. Parasites Vectors.

[52] Trotz-William LA, Trees AJ (2003). Systematic review of the distribution of the major vector-borne parasitic infections in dogs and cats in Europe. Vet Rec.

[53] Genchi C, Rinaldi L, Mortarino M, Genchi M, Cringoli G (2009). Climate and *Dirofilaria* infection in Europe. Vet Parasitol.

[54] Stangl M, Formayer H, Hofstätter M, Orlik A, Andre K, Hiebl J, et al. KlimaStatusBericht 2018. Vienna: Climate Change Centre AUSTRIA; 2019.https://ccca.ac.at/fileadmin/00_DokumenteHauptmenue/02_Klimawissen/Klimastatusbericht/Klimastatusbericht_%C3%96_2018_20190502_Printversion.pdf. Accessed 22 Oct 2020.

[55] Genchi C, Mortarino M, Rinaldi L, Cringoli G, Traldi G, Genchi M (2011). Changing climate and changing vector-borne disease distribution: The example of *Dirofilaria* in Europe. Vet Parasitol.

[56] Pampiglione S, Rivasi F (2000). Human dirofilariasis due to Dirofilaria (Nochtiella) repens: an update of world literature from 1995 to 2000. Parassitologia.

[57] Chandrasena TGAN, Premaratna R, Mallawaarachchi CH, Gunawardena NK, Gunathilaka PADHN, Abeyewickrama WY (2019). The diversity of human *Dirofilariasis* in Western Sri Lanka. Biomed Res Int.

[58] Dissanaike AS, Abeyewickreme W, Wijesundera MD, Weerasooriya MV, Ismail MM (1997). Human dirofilariasis caused by *Dirofilaria* (Nochtiella) repens in Sri Lanka. Parassitologia.

[59] European Society of Dirofilariosis and Angiostrongylosis (ESDA). Guidelines for clinical management of canine heartworm disease. 2017. https://www.esda.vet/wp-content/uploads/2017/11/GUIDELINES-FOR-CLINICAL-MANAGEMENT-OF-CANINE-HEARTWORM-DISEASE.pdf. Accessed 28 Sept 2020.

[60] Nelson CT, McCall J, Rubin SB, Buzhardt LF, Dorion DW, Graham W (2005). 2005 guidelines for the diagnosis, prevention and management of Heartworm (*Dirofilaria immitis*) infection in dogs. Vet Parasitol.

[61] Intergovernmental Panel on Climate Change (IPCC). Climate change 2014: Synthesis report. In: Contribution of Working Groups I, II and III to the Fifth Assessment Report of the Intergovernmental Panel on Climate Change; 2014. Geneva: IPCC.

