# Supplementary material for: Human dirofilariosis in Austria: the past, the present, the future
Source: Parasit Vectors. 2021 Apr 29;14:227. doi: 10.1186/s13071-021-04696-4 (PMC8082911; doi:10.1186/s13071-021-04696-4)
Supplement: Supplementary file 1 — Additional file 1: Table S1. Confirmed cases of human dirofilariosis in Austria. [file 13071_2021_4696_MOESM1_ESM.docx]

**Table S1.** Confirmed cases of human dirofilariasis in Austria.

| **Case** | **Year of diagnosis** | **Gender** | **Age** | **Localization, site** | **Histology** | **PCR** | **Antifilarial antibodies** | **Eosinophilia** | **Travel history** | **Dirofilaria species** | **Reference** |
| --- | --- | --- | --- | --- | --- | --- | --- | --- | --- | --- | --- |
| 1 | 1978 | F | 39 | thigh, upper leg; left | adult |  |  |  | Greece, Italy | *D. repens*  (7 cm) | [14] |
| 2 | 1989 | F | 27 | eyelid; left | adult |  |  | - | East Asia | *D. repens*  (8 cm) | [21] |
| 3 | 1992 | M | 36 | head/occipital; right | adult |  |  |  | Greece, Italy, Hungary | *D. repens*  (9 cm) | [22] |
| 4 | 1995 | M | 45 | trunk/axilla; right | adult |  | + | - | Bahrain, Greece | *D. repens* | [23] |
| 5 | 1996 | M | 45 | leg; left | adult + larvae |  |  |  | unknown | *D. repens* | [22] |
| 6 | 1996 | M | 35 | epididymis; left | adult |  | + | + (10%) | France, Italy, Portugal | *D. repens* | [26] |
| 7 | 1997 | F | 43 | shoulder; right | adult |  | - |  | Bosnia | *D. repens* | [22] |
| 8 | 1998 | F | 61 | orbital cavity; right side | adult |  | - | - | Greece, Italy | *D. repens*  (12 cm, female) | [24] |
| 9 | 1998 | M | 24 | inguinal area; right side | adult |  | + | + (8%) | Albania, Slovenia, Croatia | *D. repens* | [22] |
| 10 | 1998 | F | 44 | chest/axilla; left | adult |  | + |  | Greece, Spain | *D. repens* | [22] |
| 11 | 1998 | M | 43 | lumbar area | adult |  | + |  | Greece, Malta, Portugal, Italy, France, United Kingdom, The Netherlands, Switzerland | *D. repens* | [22] |
| 12 | 1999 | M | 4 | neck/back |  |  | + | + (10%) | Italy | *D. repens* | [25] |
| 13 | 2000 | M | 59 | cheek; right |  |  | + |  | unknown | *D. repens* | [25] |
| 14 | 2000 | F | 37 | chest; right | adult |  | + | + (14%) | Spain, Turkey | *D. repens* | [22] |
| 15 | 2001 | M | 11 | inguinal area |  |  | + |  | Indonesia | unspecified | [25] |
| 16 | 2001 | F | 60 | eye | not specified |  | + |  | unknown | unspecified | [25] |
| 17 | 2002 | M | 42 | arm; right | adult |  | + | + (6%) | Ethiopia, Ghana | *D. repens* | [25] |
| 18 | 2003 | M | 61 | skin; multiple nodules |  |  | + |  | Peru | *D. repens* | [25] |
| 19 | 2003 | M | 54 | inguinal area; left |  |  | + |  | unknown | unspecified | [25] |
| 20 | 2006 | F | 34 | palm; right | adult | + | + |  | none (autochthonous) | *D. repens* | [15] |
| 21 | 2008 | F | 62 | cheek/oral mucosa; right | adult | + | + |  | Hungary | *D. repens* | [28] |
| 22 | 2009 | M | 61 | epididymis; left | adult | + | - | + (9%) | Namibia | *D. repens* | [28] |
| 23 | 2009 | F | 52 | cheek; right | adult | + |  | - | Italy, Croatia, Greece, Republic of China | *D. repens* | [27] |
| 24 | 2011 | F | 45 | subconjunctival; left | adult | + |  |  | Bosnia | *D. repens*  (11 mm) | [29] |
| 25 | 2011 | F | 65 | lumbar region | adult | + |  |  | East Asia, Sri Lanka, India | *D. repens* | [25] |
| 26 | 2011 | F | 49 | head/temporal | adult |  |  |  | Croatia, Serbia | *D. repens* | [25] |
| 27 | 2012 | M | 53 | orbital cavity; right | adult | + |  | + (6.8%) | Italy, Hungary, Croatia, The Netherlands | *D. repens* | [25] |
| 28 | 2012 | F | 38 | subconjunctival; left | adult | + | - | - | India, Israel, United Arab Emirates | *D. hongkongensis* (13 cm, non-gravid female) | [17] |
| 29 | 2014 | M | 75 | umbilical | adult | + |  | + (24.9%) | Hungary | *D. repens* | [25] |
| 30 | 2014 | M | 50 | inguinal area; right | adult | + |  |  | Greece | *D. repens* | [25] |
| 31 | 2015 | F | 52 | submandibular; left |  | + |  |  | Indonesia | *D. repens* |  |
| 32 | 2017 | F | 36 | arm; right | adult | + |  | - | Thailand | *D. repens* | [30] |
| 33 | 2018 | M | 52 | leg; left |  | + | - |  | Hungary, Italy, Dominican Republic | *D. repens* |  |
| 34 | 2018 | M | 61 | eyelid; left |  | + |  |  | Serbia | *D. repens* |  |
| 35 | 2018 | F | 56 | blood, retromandibular area; right | adult, larvae | + | + | + (27%) | India, Sri Lanka, Greece | *D. repens* | [6] |
| 36 | 2019 | M | 50 | blood | larvae | + | + |  | Maldives | *D. repens* |  |
| 37 | 2019 | M | 56 | subconjunctival |  | + |  |  | Serbia | *D. repens* |  |
| 38 | 2019 | F | 64 | eyelid; left | adult | + | + | - | Bosnia | *D. repens*  (12 cm, non-gravid female) |  |
| 39 | 2020 | F | 56 | chest; left | adult | + |  |  | Croatia | *D. repens* |  |

*Abbreviations:* D., Dirofilaria; PCR, Polymerase chain reaction; F, female; M: male
